# High-Pressure Adsorption of CO_2_ and CH_4_ on Biochar—A Cost-Effective Sorbent for In Situ Applications

**DOI:** 10.3390/ma16031266

**Published:** 2023-02-02

**Authors:** Marcin Lutyński, Jan Kielar, Dawid Gajda, Marcel Mikeska, Jan Najser

**Affiliations:** 1 Faculty of Mining, Safety Engineering and Industrial Automation, Silesian University of Technology, Akademicka 2, 44-100 Gliwice, Poland; 2 Centre for Energy and Environmental Technologies, VSB-Technical University of Ostrava, 17. Listopadu 15, 70800 Ostrava, Czech Republic; 3 Institute of Meteorology and Water Management—National Research Institute, Podleśna 61, 01-673 Warszawa, Poland

**Keywords:** carbon capture, biochar, physical adsorption, carbon dioxide, methane

## Abstract

The search for an effective, cost-efficient, and selective sorbent for CO_2_ capture technologies has been a focus of research in recent years. Many technologies allow efficient separation of CO_2_ from industrial gases; however, most of them (particularly amine absorption) are very energy-intensive processes not only from the point of view of operation but also solvent production. The aim of this study was to determine CO_2_ and CH_4_ sorption capacity of pyrolyzed spruce wood under a wide range of pressures for application as an effective adsorbent for gas separation technology such as Pressure Swing Adsorption (PSA) or Temperature Swing Adsorption (TSA). The idea behind this study was to reduce the carbon footprint related to the transport and manufacturing of sorbent for the separation unit by replacing it with a material that is the direct product of pyrolysis. The results show that pyrolyzed spruce wood has a considerable sorption capacity and selectivity towards CO_2_ and CH_4_. Excess sorption capacity reached 1.4 mmol·g^−1^ for methane and 2.4 mmol·g^−1^ for carbon dioxide. The calculated absolute sorption capacity was 1.75 mmol·g^−1^ at 12.6 MPa for methane and 2.7 mmol·g^−1^ at 4.7 MPa for carbon dioxide. The isotherms follow I type isotherm which is typical for microporous adsorbents.

## 1. Introduction

The problem of greenhouse gas emission reduction has been the focus of research for the last 30 years. Global warming caused by anthropogenic emissions of gases such as carbon dioxide (CO_2_), methane (CH_4_), and nitrous oxide (N_2_O) is a serious problem in industrialized countries [1]. In the IPCC report from 2022, it was highlighted that the temperature will increase by 1.5 °C above pre-industrial levels in the next 20 years and only radical reductions in carbon emissions from now will help to avoid serious environmental impacts [2]. There are many technologies that could be deployed to decrease particularly CO_2_ emissions from power generation and industry. Among them, Carbon Capture Utilization and Storage (CCUS) is the most promising one and the study [3] suggests that that the “Golden Age” of CCUS deployment will be from 2040 to 2060 across the world. Currently, there are many technologies that allow efficient separation of CO_2_ from industrial gases. The most mature carbon capture technology is absorption technology where organic or inorganic sorbents (mostly amines and particularly monoethyloamine (MEA)), dimethyl glycol ether, and others are used as absorbents; other technologies include membrane separation or cryogenic processes [4]. The absorption technology, however, is a very energy-intensive process both from the point of view of operation and solvent production. The environmental impact of amine absorption is considerably large when taking into account solvent production, use, and regeneration. The production of MEA generates significant CO_2_ emissions due to the Haber–Bosch process [5,6]. The regeneration of the solvent after the absorption contributes to indirect CO_2_ emissions. Estimated energy intensity of the MEA absorption technology is approximately 3.8 MJ·kg^−1^ of CO_2_ captured. Another solution is the adsorption separation technology where the mixture separation process works on the principle of different adsorption/desorption properties of the constituents of the mixture. The main physical adsorption technologies are: Pressure/Vacuum Swing Adsorption (PSA/VSA) and Temperature Swing Adsorption (TSA). For the PSA/VSA process, the amount of energy required is somewhat lower than for absorption technologies, i.e., 1.18–3.2 MJ·kg^−1^ of CO_2_ captured depending on the solution. The major problem of the PSA method is the efficiency of capture, the quality of CO_2_, and the size of the unit. For PSA, typical CO_2_ recovery is 80–98% with CO_2_ purity of 90–96%, whereas for absorption methods, typical CO_2_ recovery is 95% and 98–100%, respectively [7,8]. Nevertheless, adsorption has a major advantage with regard to the ease of adsorbent regeneration by thermal or pressure changes.

In case of adsorption technologies, the main issue is the adsorbent that is used for gas separation. There are many types of adsorbents used in PSA/VSA technologies, i.e., carbon based materials (activated carbons), porous materials such as zeolites or silica gels, amine-modified structured sorbents, metal–organic frameworks (MOFs), covalent organic frameworks (COFs), microporous polymers, and clay minerals [6,9,10,11,12]. There are certain desirable criteria for the selection of adsorbents: considerable CO_2_ sorption capacity; adsorption selectivity towards CO_2_ in relation to other compounds in the mixture (particularly N_2_ in the case of flue gas and CH_4_ for pyrolytic gas); high regeneration potential; thermal, chemical, and mechanical stability; low cost of manufacturing; and availability and low environmental/carbon footprint of sorbent production or modification. In recent years, with the development of carbon footprint calculation tools and the implementation of the circular economy approach, the last two features are of utmost importance. The process of adsorbent production may be complex and energy intensive but pyrogenic carbonaceous adsorbents such as carbonized biomass, charcoal, and other low-cost carbon materials can be considered as a cost-effective solution [13,14]. Particularly, biochar can be considered as a cheap, effective, and stable adsorption material in comparison with other carbonaceous sorbents such as activated carbon, graphene, and carbon fibers [15]. Biochar in comparison with, e.g., zeolites synthesized from fly ashes has a considerable CO_2_ sorption capacity of 0.38–2.31 mmol·g^−1^ [16,17]. For example, wheat bran (agricultural biomass) combustion experiments provided fly ash and bottom ash fractions which were further processed into pellets; the maximum adsorption capacity when pelletized was 0.07 mmol CO_2_/g, and non-pelletized, 0.06 mmol CO_2_/g (25 °C), and at the temperature of 100°C the maximum sorption capacity dropped to approximately 0.024 mmol of CO_2_/g [18]. A study of CO_2_ adsorption on functionalized straw burning ashes [19] considered different additives to biomass such as halloysite and kaolinite. The results show that sorption capacity was in the range of 0.132 mmol·g^−1^ (pure straw biomass) to 0.321 mmol·g^−1^ (straw biomass with 4 wt. % kaolinite). There are also examples of conversion of palm-pruning leaves or co-pyrolysis of biomass with lubricating oil to obtain effective sorbents [20,21].

The aim of this study was to determine CO_2_ and CH_4_ sorption capacity of pyrolyzed spruce wood in a broad range of pressures for application as an effective adsorbent for gas separation technology such as PSA or TSA. The idea behind this study was to reduce the carbon footprint related to the transport and manufacturing of sorbent for the separation unit by replacing it with a material that is the direct product of pyrolysis. This allows cost-effective sorbent manufacturing in situ that can be used for purification of pyrolytic gas and separation of CO_2_. This approach is in line with the idea of a circular economy since it reduces waste generation, carbon footprint, and environmental pollution that is caused by sorbent manufacturing, processing, and transport. Similar to the approach in this case, the product of pyrolysis can be directly used for gas treatment in CO_2_ separation units.

## 2. Materials and Methods

**Figure 1:**
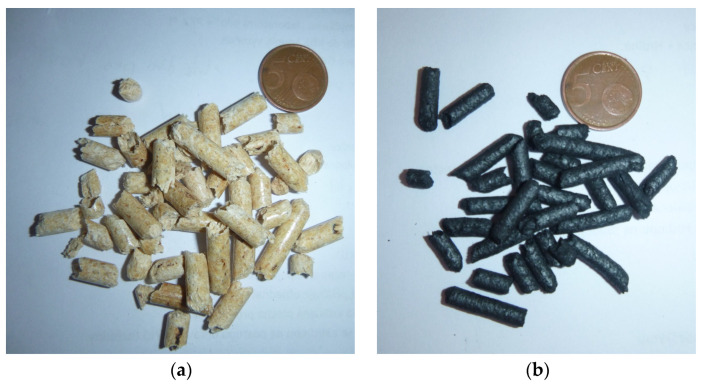
Adsorption test sample of spruce wood: before (**a**) and after (**b**) pyrolysis.

As an adsorbent, the pyrolyzed white pellets (commercial signature A1) of spruce wood manufactured by BIOMAC Energy [22] from Czech Republic were used (Figure 1). Pellets have a diameter of 6 mm, length of 10 ÷ 20 mm, and a net calorific value of 18.8 MJ·kg^−1^ [22]. The elemental analysis was determined by the elemental analyzer CHSN628, the gross analysis by the TGA analyzer TGA 701, and the heating value with the calorimeter AC 600. All analyzers are manufactured by LECO Corporation, St. Joseph, MI, USA. Spruce wood has a high content of natural resin which increases its heating value. Since the content of ash, chlorine, and sulphur are relatively low, the low bulk density increases the burn speed of the material.

Properties of spruce wood, used in the pyrolysis and then in the adsorption tests, are shown in Table 1.

The chemical composition of investigated wood material is presented in Table 2.

**Figure 2:**
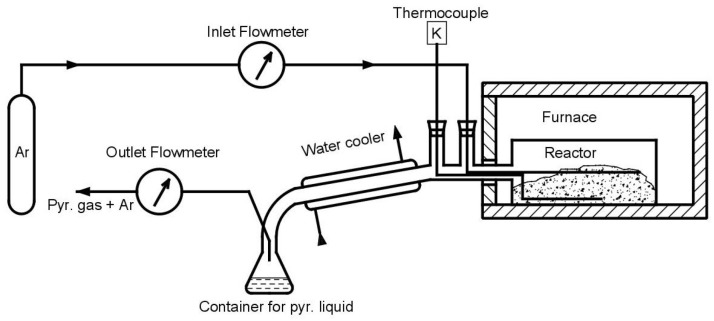
Scheme of laboratory pyrolysis unit.

Pyrolysis was carried out in a laboratory pyrolysis unit as shown in Figure 2. The major components of the device are made of laboratory glass. Argon, as an inert gas (SIAD Argon 5.0) is injected into the reactor chamber from the pressure cylinder, using a pressure reducing valve. It then flows through an AALBORG model GFC17 flow meter. The argon flow rate was 1 dm^3^·min^−1^ for the duration of the experiment. The gas enters the reactor through the capillary. A K-type thermocouple is also placed in the reactor to monitor the actual temperature inside the reactor. The fuel for pyrolysis is placed in the reactor. The entire reactor is located in the furnace, equipped with a process controller. The products of the pyrolysis process exit the reactor through a water cooler, which allows cooling and condensation of the vapors. The liquid is collected in a flask and gas exits the process through a drum flow meter. The gas is further removed for analysis and subsequent disposal.

**Figure 3:**
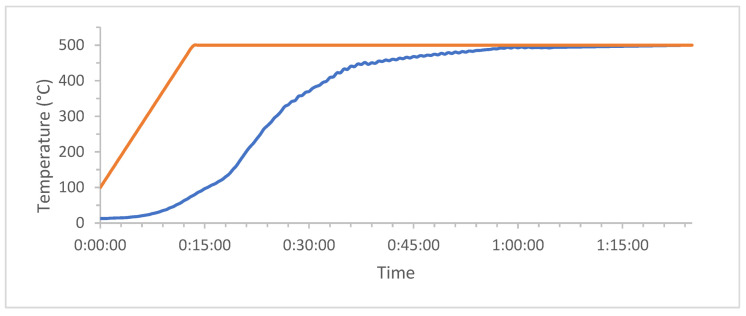
Temperature curve of the pyrolysis process (orange—temperature in furnace; blue—temperature in reactor).

The samples of spruce wood pellets were loaded into the reactor which was then sealed. Subsequently, the furnace was heated from 100 °C with a temperature ramp of 0.5 °C·s^−1^ up to a temperature of 500 °C. The temperature was subsequently maintained in the reactor for 1 h. The heating in the pyrolysis reactor was controlled by the furnace temperature program (Figure 3). The reactor is heated at a slower rate due to the heat transfer through the reactor wall, the specific heat capacity of the pyrolyzed material, and the pyrolysis process itself. The pyrolysis was completed when the flow rates at the inlet and outlet flow meters were similar.

The samples used for the adsorption tests after the pyrolysis process are black in color, brittle, and contain micropores. The granulometry and shape of the samples (see Figure 1) remain unchanged due to pyrolysis. The mass balance of pyrolysis was: char: 70%_mass_, pyrolysis oil: 26%_mass_, and gas: 4%_mass_.

**Figure 4:**
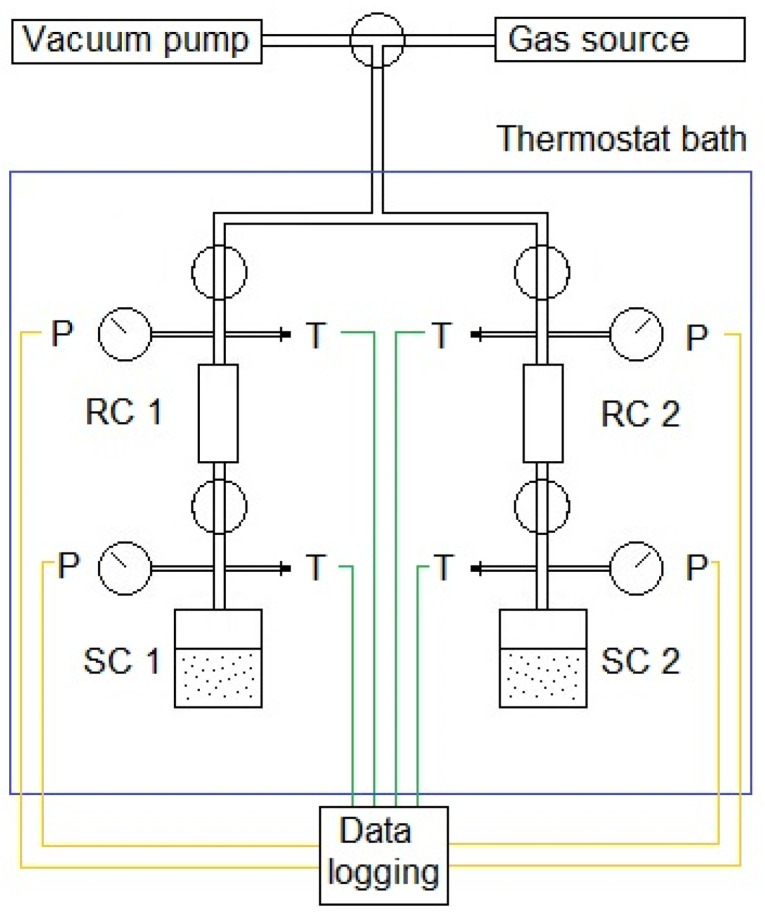
Scheme of laboratory sorption setup used in the study (SC—sample cell; RC—reference cell).

Adsorption tests were performed using the volumetric sorption method. The setup consists of two parallel and independent lines providing the possibility to test two separate samples in the same conditions using the same gas. Two parallel tests were carried out in order to verify the obtained results and check for inconsistencies. The experiments were performed at a constant temperature and the setup was immersed in a water bath, maintaining a stable temperature of 40 °C. The water bath also helps to detect any leaks, which have a significant influence on the final results. The setup consists of 3 volume sections: the first is common for both of the lines and is used for vacuuming and injection of the gas. The second section, separate for each line, is a reference volume (RC—reference cell), with known volume, which enables calculation of the amount of gas supplied to the setup. The last sector is a sample cell (SC), including a container, where the tested sample is placed. All mentioned sectors are separated with valves. The setup is equipped with pressure transducers (Keller Druck X33) with an accuracy of 0.001 bar and connected to the data collector with constant data logging (CCM software v1.0). The scheme of the sorption setup is shown in Figure 4.

Samples were placed in the sample cells and the setup was assembled and checked for leaks. First, any remaining gas was removed from the setup by vacuuming for approximately 8 h. Then, helium was injected into the reference cell. After stabilizing the pressure and temperature, the valve between the reference cell and the sample cell was opened, allowing helium to flow into the sample cell. After recording the pressure drop, it was possible to determine the void of the volume of the sample cell. Then, the valve between the reference cell and sample cell was closed and an additional amount of helium was pumped into the reference cell. Then, the valve was again opened, increasing pressure in the entire setup. The process was repeated three times and the determined volume of the measuring cell was calculated as an arithmetic mean of all steps. The calculations were made on the basis of the McCarthy [23] equation of state. Helium was used for determining the measuring cell volume as a non-adsorbing gas.

**Figure 5:**
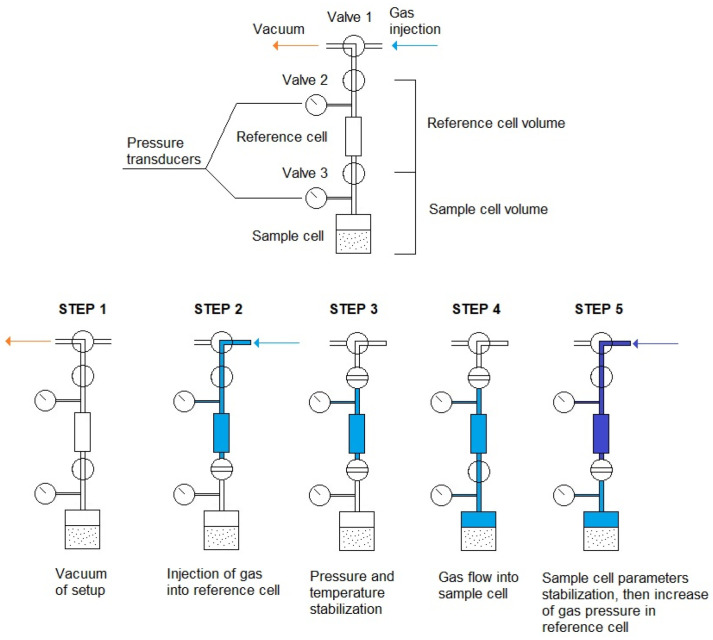
Sorption test procedure.

After determining the volume of the measuring cell, the setup was vacuumed and filled with carbon dioxide. After filling the reference cell and stabilization of conditions, the valve between the reference cell and the sample cell was opened and carbon dioxide was passed into the tank with the tested sample. The physical sorption process then occurred on the surface of the samples. A pressure drop was observed over time and when the equilibrium was reached the sorption rate could be calculated. The highest sorption rate occurred in the first minutes of the test, while determination of the full sorption capacity took up to a few hours. The next step of closing the sample cell valve, raising the gas pressure in the reference cell, stabilizing conditions and opening the valve, and then letting the next portion of gas flow into the sample cell, was conducted one day after the previous step. A total number of 5 steps were conducted up to the pressure of 10 MPa. The same test procedure was conducted for methane. The gas injection procedure is shown in Figure 5.

Reference cell volumes were determined for each line of the setup, and were 151.382 cm^3^ and 154.039 cm^3^, respectively. The mass of adsorbent (pyrolyzed spruce wood) placed in the sample cell was 73.5 g and 59.7 g in line 1 and 2 of the setup, respectively.

## 3. Results and Discussion

**Figure 6:**
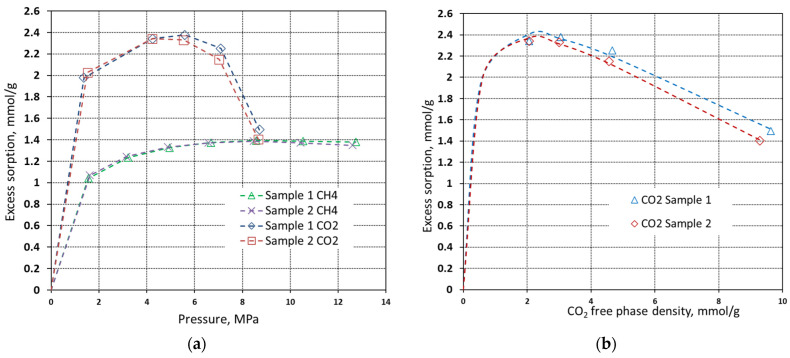
Carbon dioxide and methane sorption isotherms of pyrolyzed spruce wood measured at 40 °C (**a**) and plot of carbon dioxide excess sorption isotherms plotted against CO_2_ free phase density with fitted Langmuir model (dashed line) (**b**).

A total of 2 different samples made of the same material were investigated for both carbon dioxide and methane adsorption at the temperature of 40 °C. The pressure range was from 0 MPa to almost 13 MPa. Sorption experiments had seven steps for methane and five for CO_2_. The results of the tests for both carbon dioxide and methane are presented as the adsorbed volume (in mmol·g^−1^) versus pressure in Figure 6a. The results of CO_2_ excess sorption capacity were also plotted versus the CO_2_ free phase density (see Figure 6b) since the supercritical region shows a clear decrease in excess sorption capacity for CO_2_. A three-parameter Langmuir model was fitted to empirical data points [24]. In this case, the Langmuir equation is modified to take into account density rather than pressure, whereas the adsorbed phase density is accounted as the “free” variable:

(1) $$m_{exc}^{}=\frac{m_{a}\cdot \rho_{g}}{b_{v}+\rho_{g}}\left(1-\frac{\rho_{g}}{\rho_{a}}\right)$$

In Equation (1) *m_exc_* is the excess sorption capacity, *m_a_* is the maximum monolayer capacity, *b_v_* is the Langmuir equilibrium constant (the inverse of Langmuir pressure), *ρ_g_* is the free phase gas density, and *ρ_a_* is the adsorbed phase gas density. In this case, the adsorbed phase density can be determined for each measurement, whereas the Langmuir isotherm well matches the volume versus CO_2_ density curve. In order to fit the model to the empirical data, minimization of the residual sum of squares was carried out in EXCEL solver using the goal-seek function.

Isotherms show that the maximum excess sorption capacity of methane is 1.4 mmol·g^−1^, while the sorption capacity of carbon dioxide reaches 2.4 mmol·g^−1^ of sorbent. To assess the true adsorption capacity of sorbent it is necessary to present data as the absolute sorption capacity where it refers to the actual number of molecules present in the pores. The relation between the excess sorption capacity and absolute sorption capacity can be expressed with Equation (2).

(2) $$m_{abs}=\frac{m_{exc}}{\left(1-\frac{\rho_{g}}{\rho_{a}}\right)}$$

**Figure 7:**
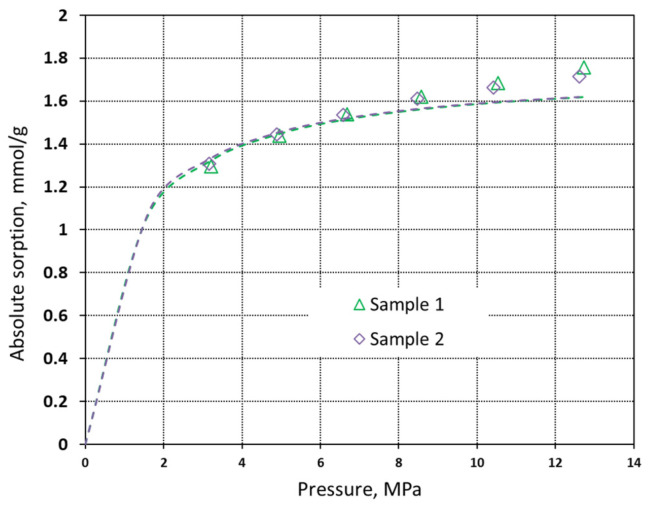
Absolute sorption isotherms of methane on pyrolyzed spruce wood at 40 °C with the Langmuir model fitted (dashed line).

**Figure 8:**
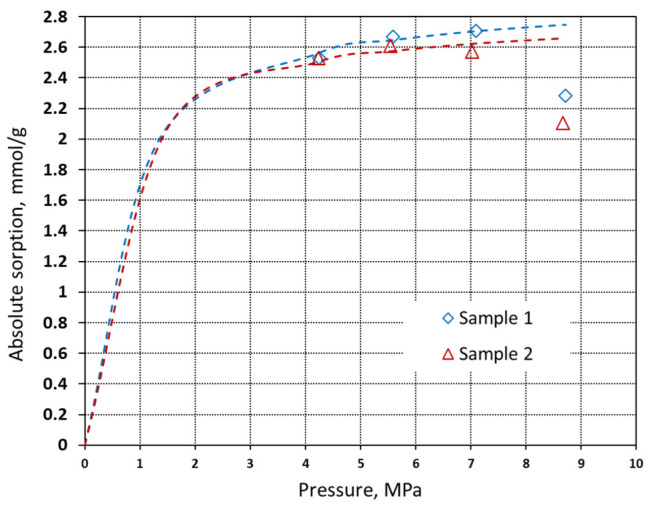
Absolute sorption isotherms of carbon dioxide on pyrolyzed spruce wood at 40 °C with the Langmuir model fitted (dashed line).

In this relation, the adsorbed phase density (*ρ_a_*) is a constant which cannot be determined experimentally. In this study, the adsorbed phase density was assumed as a liquid density at boiling point and is equal to 27.881 mmol·dm^−3^ for CO_2_ and 26.24 mmol·dm^−3^ for methane. The absolute sorption isotherms are presented in Figure 7 and Figure 8.

The absolute sorption data show that the maximum measured methane sorption capacity is approximately 1.75 mmol·g^−1^ at 12.6 MPa, whereas for CO_2_ the maximum sorption capacity is 2.7 mmol·g^−1^ at 4.7 MPa. In Table 3, the predicted Langmuir parameters are presented. The calculated adsorbed phase density for CO_2_ using the three-parameter Langmuir model was in the range of 18.0–19.9 mmol·dm^−3^.

The obtained results show that pyrolyzed spruce pellets have a considerable sorption capacity towards gases. As was expected, the sorption capacity of CO_2_ is almost twice as high as that of CH_4_ as is observed in many microporous adsorbents. The excess sorption curve follows I type isotherm in accordance with the new classification of isotherms for physical adsorption of gases on solids [25]. The maximum sorption capacities are approximately three times lower than those of activated carbons [26,27,28,29]. However, they are comparable to bituminous coals, anthracites, and zeolites synthesized from fly ashes [30,31,32]. Sorption properties and relatively high carbon content make it possible to be used either as a direct gas sorbent which can be disposed easily or a precursor of activated carbons.

## 4. Conclusions

The main aim of this study was an initial assessment of pyrolyzed spruce pellet (biochar) sorption capacity towards CO_2_ and CH_4_. The lower cost of manufacturing of this material is related to the fact that the process of carbonatization is a part of the pyrolytic process. Even without the activation stage, the sorption capacity of this material was relatively high. Based on the study, it was revealed that this material has a considerable sorption capacity and selectivity towards gases used in the study. Excess sorption capacity reached 1,4 mmol·g^−1^ for methane and 2.4 mmol·g^−1^ for carbon dioxide. The calculated absolute sorption capacity was 1.75 mmol·g^−1^ at 12.6 MPa for methane and 2.7 mmol·g^−1^ at 4.7 MPa for carbon dioxide. Isotherms follow I type isotherm typical for microporous adsorbents. Nevertheless, further studies related to micropore structure of this sorbent, microstructural analysis, and degradation during sorption/desorption cycles are needed to assess its suitability as a gas sorbent. A more detailed study is on the way as these results are an initial trial to assess the sorption capacity. There is room for improvement in terms of surface properties of this sorbent. It should be noted that sorption capacities shown in the study are for the raw material without any activation process nor chemical treatment. Future studies should focus on such activities and assess whether the cost-efficiency of such an approach will be effective.

## Figures and Tables

**Table 1:** Properties of investigated raw material.

Property	Symbol, Unit	Value
Ash content ^1^	A_d_, %_mass_	0.53
Volatile matter ^2^	V_daf_, %_mass_	83.7
Carbon	C_daf_, %_mass_	50.3
Hydrogen	H_daf_, %_mass_	6.16
Oxygen	O_daf,_ %_mass_	43.4
Nitrogen	N_daf_, %_mass_	0.12
Chlorine	Cl_daf_, %_mass_	<0.01
Sulphur	S_d_, %_mass_	0.01
Heating value	Q_p, net, d_, MJ·kg^−1^	18.8
Tannins	%_mass_	8.75 ± 0.12
Lignin	%_mass_	23.29 ± 0.02
Resinous substances	%_mass_	3.49 ± 0.14
Holocellulose	%_mass_	54.92
Deformation temperature	°C	1044
Softening temperature	°C	1052
Melting point	°C	1257
Creep temperature	°C	1264

^1^ d—anhydrous state of the sample. ^2^ daf—dry ash free state of the sample.

**Table 2:** Chemical composition of investigated raw material (%_mass_).

Oxides/Element	Value
P_2_O_5_	3.53
Al_2_O_3_	2.66
Na_2_O	0.59
SO_3_	2.15
SiO_2_	8.50
CaO	43.50
K_2_O	9.24
Fe_2_O_3_	1.53
MgO	6.68
TiO	0.18
MnO	3.780
Cl	0.490
Pb	0.023
Cd	0.001
Cu	0.038
Hg	<0.001
Cr	0.120
Ni	0.780
V	BDL
Zn	0.140

**Table 3:** Langmuir parameters calculated for the absolute sorption.

	Sample 1	Sample 2	Sample 1	Sample 2
Adsorbate	CH_4_		CO_2_	
m_a_, mmol·g^−1^	1.75	1.74	2.95	2.82
b_v_, mol·dm^−3^	0.96	1.01	1.57	1.85
R^2^	0.996	0.997	0.995	0.983

## Data Availability

The data presented in this study are is contained within the article.
